# Pigmented Birthmarks and Spinal Neurofibromas in *KRAS* Mosaicism—Not to Be Confused With NF1

**DOI:** 10.1111/pde.16018

**Published:** 2025-07-19

**Authors:** Karina M. Forde, Nicole Knöpfel, Ulrike Loebel, Veronica A. Kinsler

**Affiliations:** ^1^ Paediatric Dermatology Great Ormond Street Hospital for Children NHS Foundation Trust London UK; ^2^ Paediatric Dermatology, Addenbrooke's Hospital Cambridge University Hospital NHS Trust UK; ^3^ UCL GOS Institute of Child Health London UK; ^4^ Mosaicism and Precision Medicine Laboratory The Francis Crick Institute London UK; ^5^ Neuroradiology Unit, Department of Radiology Great Ormond Street Hospital NHS Foundation Trust London UK

**Keywords:** mosaicism, neurofibromatosis type 1, phakomatosis pigmentokeratotica, spinal neurofibromas

## Abstract

We report a child presenting with pigmentary skin lesions and spinal neurofibromas who was diagnosed molecularly with *KRAS* mosaicism. We review the previous literature of two cases of congenital skin lesions and neurofibromas and spinal nerve root hypertrophy caused by *KRAS* variants and highlight this presentation as an important differential diagnosis for neurofibromatosis.

## 1 Introduction

Mosaicism is increasingly being discovered as the cause of undiagnosed neurocutaneous phenotypes. These disorders are caused by a post‐zygotic single cell pathogenic variant arising at some point during embryogenesis or fetal development, leading to a clonal genetic disease in only part of the individual. As the variant can arise at any point and in any cell lineage, the phenotype of these diseases varies widely, and this new understanding is leading to reclassification of disease spectra, rather than multiple separate clinical diagnoses [1]. It is important to diagnose these diseases genetically, which requires investigation of tissue rather than blood, as it can have implications for cancer risk as well as for potential transmission to offspring [1].

### 1.1 Case Report

**FIGURE 1:**
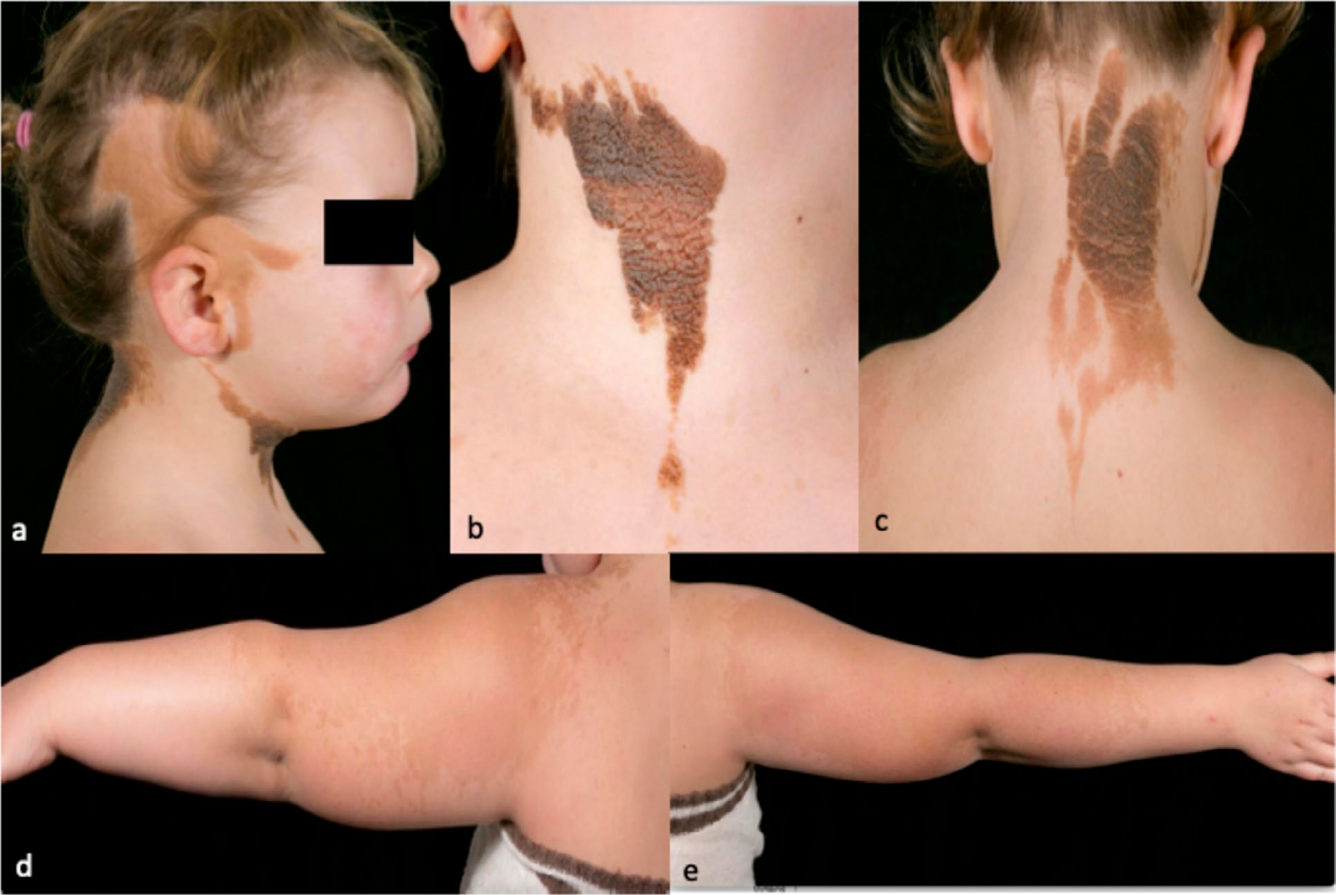
(a) Blaschkolinear sebaceous epidermal nevus on the right temperoparietal scalp and a faint hyperpigmented macule on the right cheek, anterior to the epidermal nevus. (b, c) Pigmented Blaschkolinear keratinocytic epidermal nevus on the anterior and posterior neck, extending onto the upper back. (d, e) Macular hyperpigmentation on the extensor aspects of both shoulders, upper and lower arms, with a few small (1 mm) superimposed hyperpigmented macules within the right lesion (e) but not the left (d).

A 4‐year‐old girl was referred with birthmarks and a coloboma. Clinical examination revealed a right‐sided Blaschkolinear epidermal nevus, sebaceous in nature on the temporoparietal scalp in association with alopecia, and pigmented and keratinocytic on the anterior and posterior neck and upper back (Figure 1a–c). She had two large patches of discrete macular hyperpigmentation on the extensor surfaces of both upper limbs extending from above both shoulders to below the elbows, which on the right, but not the left, contained a few small (1‐mm) superimposed hyperpigmented macules (Figure 1d,e). An additional two‐centimeter hyperpigmented macule was present on the right cheek, anterior to the epidermal nevus. The skin signs were therefore compatible with phakomatosis pigmentokeratotica. Subtle facial asymmetry was noted, right slightly larger than left. Oral examination demonstrated involvement of the right oral mucous membrane by the epidermal nevus as far as the uvula.

**FIGURE 2:**
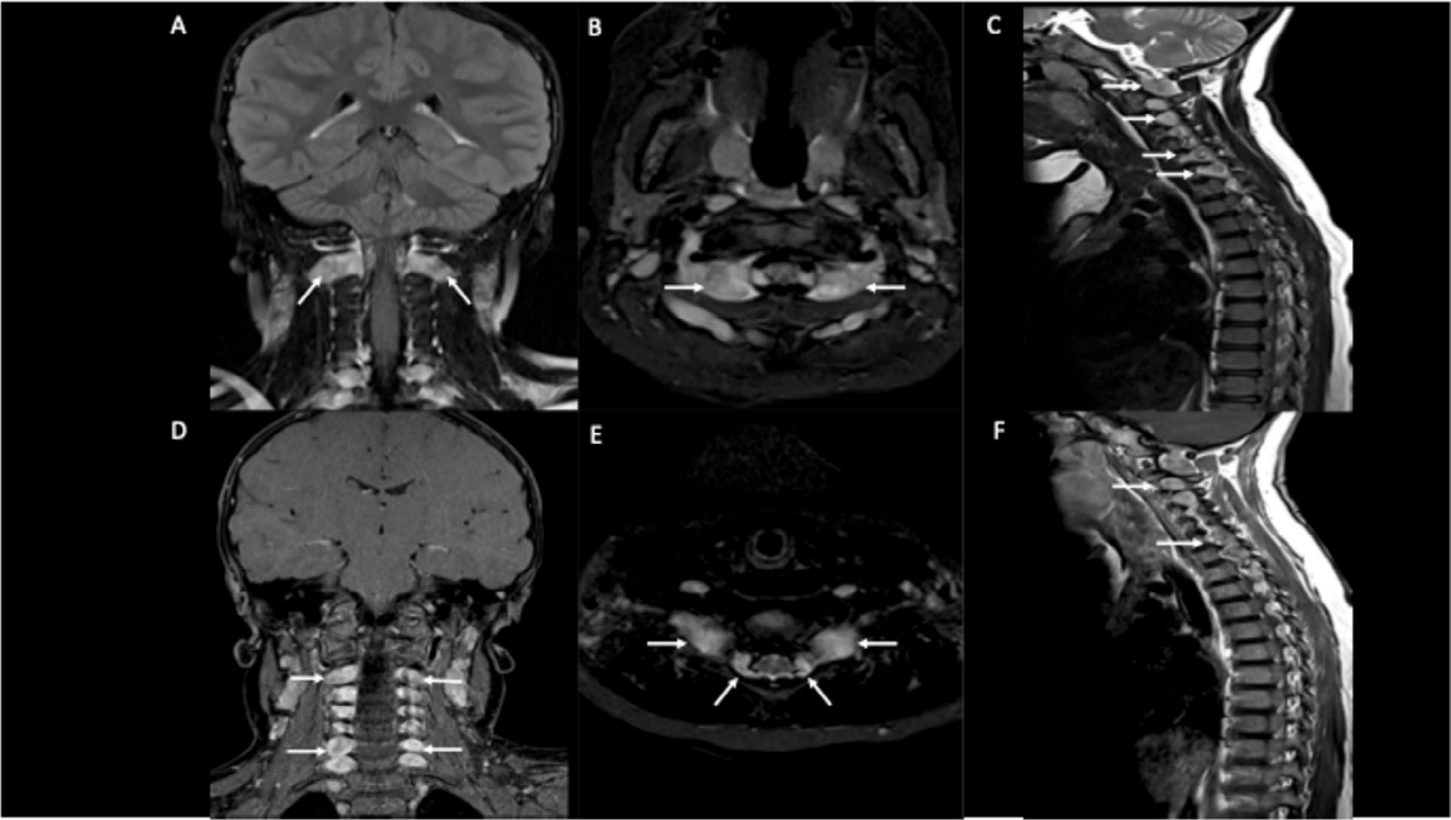
MRI of the brain and spine showing multiple masses in the bilateral neuroforamina of the cervical spine, in keeping with neurofibromas (arrowed a–f), which enhance after contrast administration (d, f). Sagittal imaging of the spine at 6 years on T2 imaging (c) and on contrast‐enhanced T1imaging (f).

Magnetic resonance imaging (MRI) of the face, undertaken due to a history of gagging and the visible epidermal naevus involvement of the uvula, showed no concerning abnormalities in the neck spaces and the gagging resolved. However, the MRI revealed bilateral cervical neurofibromas. Subsequent MRI of the whole spine showed multilevel bilateral T2‐hyperintense lesions from the cranial cervical junction to C6/C7 vertebral levels (Figure 2), consistent with cervical neurofibromas. Repeat imaging was stable after 6 years, with no symptoms thus far from the spinal lesions.

Biopsy from the epidermal nevus on the neck demonstrated acanthosis, papillomatosis, and hyperkeratosis. Targeted panel next generation sequencing performed on DNA extracted from the whole skin biopsy without cell culture identified a pathogenic variant NM_004985.5 *KRAS* c.35G>A, p.(Gly12Asp), at 39% variant allele frequency (VAF). This was undetectable in blood, confirming mosaicism.

The co‐existence of cutaneous lesions and neurofibromas often triggers investigation for neurofibromatosis 1 (NF1), particularly where the cutaneous signs include pigmented macular lesions. This case demonstrates the importance of investigation for mosaicism and in particular to consider *KRAS* mosaicism with this presentation as has been described in two previous cases this is the third description of this phenotypic association (Table 1).

**TABLE 1:** Summary of three reported cases of *KRAS* mosaicism presenting with cutaneous lesions and neurofibromas/hypertrophic neuropathy.

Patient	1 (Farschtschi et al. 2015)	2 (Dionysou M et al. 2024)	Our patient
Age/Sex	17Y, M	7Y, F	9Y, F
Cutaneous	Blaschkolinear keratinocytic epidermal nevus right upper back. Large areas of macular hyperpigmentation on upper trunk and both arms.	Blaschkolinear keratinocytic epidermal nevus left posterior neck, upper back and left flank. Epidermal nevus on scalp.	Blaschkolinear sebaceous epidermal nevus right posterior parietal scalp. Uvular epidermal nevus. Blaschkolinear keratinocytic epidermal nevus right posterior neck and upper back. Two large patches of macular hyperpigmentation on the shoulders bilaterally.
Neurological	Delayed motor development. Normal MRI brain. Cervical and thoracic tumor (C3‐T1) with histological findings of Schwann cell proliferation resembling onion bulb formation. Intraspinal lipoma C5‐C7. Transversal spinal cord syndrome with spasticity, paraparesis and pain. Proximal neuropathy. Thoracolumbar scoliosis	Diffuse spinal nerve hypertrophy involving left C1‐C7 bilateral T1‐T2, L3, L4 and sacral plexus with progressive growth causing cervical spinal cord compression. Partial complex epilepsy, well controlled. Speech delay otherwise normal neurodevelopment.	Bilateral cervical neurofibromas from C2‐C6
Ocular	Bilateral impaired vision (50%) and astigmatism		Right coloboma
Other	Thoracolumbar scoliosis. Generalized osteopenia.	Congenital heart disease (ectopic atrial tachycardia, PDA, coarctation of the aorta), Polycystic kidney disease, benign pelvic ganglioneuroma. Mild facial asymmetry (*R* > L)	Subtle facial asymmetry (*R*>L)
*KRAS* variant	c.35G > A p. (Gly12Asp) VAF 44% (skin) 49% (perineuroma) and 56% (lipoma)	c.35G>A p.(Gly12Asp) VAF not reported	c.35G>A p.(Gly12Asp) VAF 39%
Tissue samples tested	Skin, intraneural perineuroma, and lipoma	Epidermal nevus	Epidermal nevus

*Note*: L left; F female; KEN keratinocytic epidermal nevus; M male; MRI magnetic resonance imaging; PDA patent ductus arteriosus; R right; VAF, variant allele frequency; Y years.

The first reported case was a 17‐year‐old male with Blaschkolinear pigmented keratinocytic epidermal nevi involving the upper back, and segmental pattern macular hyperpigmentation on the upper trunk and both arms, consistent with phakomatosis pigmentokeratotica. A large cervical and upper thoracic intraspinal Schwann cell tumor and lipoma with cylindrical thickening of the spinal nerves and intraspinal extension presented with right‐sided compression of the spinal cord. The c.35G > A, p.(Gly12Asp) *KRAS* variant (44% VAF) was identified in affected skin, Schwann cell tumor (49%) and lipoma (56%) and was undetectable in blood. At the time of report, his neurological symptoms were progressive, and he was wheelchair‐bound [2].

The second reported case was a 7‐year‐old female with a keratinocytic epidermal nevus on the left posterior neck, upper back, and left flank, and scalp, in whom widespread spinal nerve hypertrophy was detected incidentally at the age of two. *KRAS* variant c.35G > A, p.(Gly12Asp) (no VAF) was detected in affected skin and was undetectable in blood. Radiological progression of spinal nerve root hypertrophy prompted treatment with the MEK1/2 inhibitor selumetinib. Stability of nerve root hypertrophy and flattening of the epidermal nevus was reported [3].

These three cases have all been associated with the same pathogenic variant c.35G > A, p.(Gly12Asp); however, this is the most frequent *KRAS* variant reported in human cancer [4] and the numbers here are too small to suggest the variant itself is specifically important. Similarly, it is not possible to conclude whether all three having cutaneous lesions affecting the head/neck/upper back is relevant to the chance of spinal involvement. We have reason to suspect that this type of presentation could be seen with other distributions and pathogenic variants as there is a description of due to, an 11‐year‐old boy with four café au lait macules and diffuse enlargement of the lumbosacral plexus and right sciatic nerve, tibial and peroneal nerves with a different *KRAS* pathogenic variant c.38_40dupGCG p.(G13dup) in affected skin and Schwann cells from the peripheral tibial nerve (not detected in blood) [5]. There were, however, no published skin photographs for that case, so we cannot be certain about the cutaneous phenotype.

Our patient, now aged 10, remains under surveillance. Radiological and/or clinical progression of spinal neurofibromas has been reported, but owing to the rarity and heterogeneity of mosaic disorders, the natural history cannot be accurately predicted. Should treatment be required, targeted medical management with a MEK inhibitor, as reported in one previous case [3] and as established as effective treatment for *NF‐*1‐related plexiform neurofibroma [6], could be considered. Regarding genetic counseling, many mosaic conditions are now understood to be potentially transferrable to offspring as germline diseases. This particular *KRAS* variant has thus far not been reported constitutionally in humans; however, other *KRAS* variants are reported in the germline causing cardiofacial cutaneous and Costello syndromes. Counseling must therefore be done on an individual basis with close regard for the exact variant, not only the gene.

This case highlights the phenotypic overlap between germline and mosaic neurocutaneous conditions and adds to the growing literature linking *KRAS* to spinal nerve tumors. As the management of mosaic *NF1* and mosaic *KRAS* may be different, particularly regarding tumor susceptibility and clinical genetic counseling, this case also highlights the importance of securing a molecular diagnosis. Clinicians caring for mosaic patients with dermatological findings potentially linked to *KRAS* should be vigilant to symptoms and signs suggestive of neuropathy or spinal cord compression.

## Author Contributions

Karina M. Forde contributed to the conception and design of the paper, literature review, and drafting and revision of the manuscript. Nicole Knöpfel contributed to the interpretation of the clinical course of the patient, and generation of figures. Ulrike Loebel contributed to the interpretation of the neuroimaging and generation of relevant figures. Veronica A. Kinsler provided the primary clinical care to the patient, established, and interpreted the genetic diagnosis integral to the report, and contributed to the conception, design and critical revisions of the final manuscript. All authors read and approved the final manuscript.

## Consent

Written informed consent for publication of clinical details and photographs was obtained from the guardians of the patient.

## Conflicts of Interest

The authors declare no conflicts of interest.

## Data Availability

Data sharing is not applicable to this article as no new data were created or analyzed in this study.
